# LPS independent activation of the pro-inflammatory receptor Trem1 by C/EBPε in granulocytes

**DOI:** 10.1038/srep46440

**Published:** 2017-04-25

**Authors:** Hyung C. Suh, Touati Benoukraf, Pavithra Shyamsunder, Tong Yin, Qi Cao, Jonathan Said, Stephen Lee, Ricky Lim, Henry Yang, Jacqueline Salotti, Peter F. Johnson, Vikas Madan, H. Phillip Koeffler

**Affiliations:** 1 Division of Hematology-Oncology, David Geffen School of Medicine at UCLA, Los Angeles, CA, USA; 2 Cancer Science Institute of Singapore, National University of Singapore, Singapore; 3 Division of Hematology-Oncology, Cedars-Sinai Medical Center, UCLA School of Medicine, Los Angeles, CA, USA; 4 Department of Pathology, David Geffen School of Medicine at UCLA, Los Angeles, CA, USA; 5 Eukaryotic Transcriptional Regulation Section, Mouse Cancer Genetics Program, NCI-Frederick, Frederick, MD, USA; 6 Department of Hematology-Oncology, National University Cancer Institute of Singapore (NCIS), National University Hospital, Singapore

## Abstract

C/EBPε is a critical transcriptional factor for granulocyte differentiation and function. Individuals with germline mutations of C/EBPε fail to develop normal granulocytes and suffer from repeated infections. In order to gain a global view of the transcriptional machinery regulated by C/EBPε, we performed whole-genome ChIP-Seq using mouse bone marrow cells. To complement the C/EBPε DNA binding analyses, RNA-Sequencing was done in parallel using sorted mature and immature granulocytes from WT and C/EBPε KO bone marrow. This approach led to the identification of several direct targets of C/EBPε, which are potential effectors of its role in granulocytic differentiation and function. Interestingly, *Trem1*, a gene critical to granulocyte function, was identified as a direct C/EBPε target gene. Trem1 expression overlaps very closely with expression signature of C/EBPε during hematopoietic development. Luciferase reporter and EMSA assays revealed that C/EBPε binds to the regulatory elements of *Trem1* and regulates its expression during granulocytic differentiation. In addition, we provide evidence that inflammatory stimuli (LPS) can also control the expression of Trem1 independent of C/EBPε. Overall, this study provides comprehensive profiling of the transcriptional network controlled by C/EBPε during granulopoiesis and identifies Trem1 as one of its downstream effectors involved in eliciting an immune response.

Mature granulocytes arise from a hematopoietic stem cell via a series of events that involve myeloid lineage commitment, proliferation, and differentiation. The terminal phases of granulopoiesis are marked by distinct transcriptional changes including granule formation, changes in expression of cell surface markers, and segmentation of the nucleus. Terminally differentiated granulocytes constitute a dominant portion of circulating white blood cells and make up an important component of innate immunity.

The innate immune system serves as the first line of defense against infectious and malignant diseases^1^,^2^. Immune reaction of granulocytes starts with sensing a pathogen with sensors such as pattern recognition receptors and intracellular DNA or RNA receptors^3^. The sensors provoke molecular machinery of proliferation, differentiation, migration, and egress from the bone marrow of immature granulocytes. When stimulated, granulocytes migrate to the inflammatory area, perform phagocytosis, produce degranulation and release neutrophil extracellular traps^4^. The phagocytic function of neutrophils depends on both the synthesis of cytoplasmic granules and the ability to initiate oxidative bursts, which are tightly regulated to avoid collateral damage to the host^4^. Once the inflammation is cleared, activated anti-inflammatory mechanisms counterbalance inflammation to achieve homeostasis preventing further damage to host cells^5^.

The importance of transcription factors, C/EBPε, PU.1, and Gfi-1, in the differentiation of granulocytes, has been previously described^6^,^7^,^8^,^9^,^10^. C/EBPε mRNA is highly expressed in myeloid bone marrow (BM) cells, especially at the transition from the promyelocyte to the myelocyte stage of differentiation, suggesting a critical role in the regulation of granulocyte-specific genes^11^. The granulocytes of individuals who have germline mutation of C/EBPε fail to show appropriate segmentation and phagocytosis and their granulocytes cannot make secondary granule proteins^12^,^13^. C/EBPε knock-out (KO) mice have similar defects and their granulocytes have been shown to be defective in their ability to migrate through the peritoneal membrane^14^,^15^. As a result of these defects, C/EBPε KO mice often have shorter survival secondary to bacterial infections^7^,^16^.

Human TREM1 (Triggering receptor expressed on myeloid cells-1) is a 30 kDa glycoprotein of the Ig family, and has a short cytoplasmic tail lacking any signaling motif^17^. TREM1 is mainly expressed in mature myeloid cells and is involved in activation of pro-inflammatory innate immune response to detect and eliminate pathogens efficiently^18^. TREM1 expression is upregulated by either LPS stimulation or bacterial infection via the NF*k*B signaling pathway^19^. While IL-1β, TNF-α, and MCP-1 production were inhibited by blockade of TREM1^20^, ligation of TREM1 on granulocytes induces production of IL-8, MPO, lactoferrin, and reactive oxygen species, leading to rapid degranulation of neutrophilic granules, and phagocytosis^18^,^21^. Therefore, TREM1 is closely associated with a broad spectrum of granulocytic functions.

In this study, we identify Trem1 as a novel transcriptional target of C/EBPε during granulopoiesis. We characterize a comprehensive network of transcriptional changes caused by a deficiency of C/EBPε, which possibly contribute to maturation block of granulocytes. By integrating ChIP-Seq and RNA-Seq data, we recognized several direct targets of C/EBPε, including Trem1. Our cellular and molecular studies demonstrate that C/EBPε regulates *Trem1* expression during maturation of granulocytes, independent of LPS induced inflammatory signaling.

## Results

### The transcriptomic landscape of C/EBPε KO granulocytes

Expression of C/EBPε parallels induction of terminal differentiation of granulocytes. Gr-1 and Mac-1 are markers for myeloid cells in mice, and Gr-1 expression increases as granulocytes differentiate^11^,^22^. Two distinct populations in the Gr-1^+^/Mac-1^+^ double positive cells are identified in the murine bone marrow: Gr-1int (Gr-1intermediate/Mac-1^+^) and Gr-1hi (Gr-1high/Mac-1^+^). We compared the flow-cytometry pattern of wild-type and C/EBPε KO BM cells and observed that majority of C/EBPε KO granulocytes expressed an intermediate level of the Gr-1 antigen (Gr-1int) (Supplementary Figure 1A). C/EBPε KO BM granulocytes also had less granular content as determined by the side scatter analysis (Supplementary Figure 1B). This represents a known defect in terminal differentiation of granulocytes in the C/EBPε deficient mice^7^,^14^. The morphology of this cell population was confirmed microscopically as having fewer segmented neutrophils (Supplementary Figure 1C), which is consistent with a previous observation^7^.

To identify target genes of C/EBPε in terminal differentiation of granulocytes, the two populations, Gr-1hi and Gr-1int, were sorted from both WT and C/EBPε KO BM, and the global changes in gene expression were compared using whole transcriptome sequencing (RNA-Seq) (Supplementary data 1). This allowed us to compare the transcriptomic consequences of loss of C/EBPε in two independent populations.

The comparative analysis between WT and KO BM cells revealed 917 transcripts in Gr-1int and 1,997 transcripts in Gr-1hi cells, which were differentially expressed with a FPKM log ratio of 4 fold. Among them, 394 transcripts were commonly expressed in both Gr-1int and Gr-1hi populations including well-known genes activated by C/EBPε such as cathelicidin antimicrobial peptide (*Camp*)^23^, and *Ngp*^24^. However, these transcriptomic data alone do not distinguish direct targets of C/EBPε from subsequent secondary effects. Identification of C/EBPε binding sites within gene regulatory elements is required for the characterization of its primary effectors.

### Genome-wide analysis of C/EBPε binding

To characterize the genome-wide C/EBPε binding sites, we performed ChIP-Seq in murine BM cells, which allowed us to build a comprehensive catalog of C/EBPε binding sites in hematopoietic cells. ChIP-Seq reads were mapped to the mouse reference genome build 37 (mm9), which were visualized using the UCSC Genome Browser (http://genome.ucsc.edu) as described in “Method”. Of the 40,517 bindings sites characterized for C/EBPε, 10% are located within the gene promoter region (−1 kb to +100 bp from the transcription starting site (TSS)), while the others are in exons (2%), introns (46%), 3′-UTRs (1%), transcription termination sites (TTS, 2%) and intergenic (39%) regions (Supplementary data 1, Supplementary Figure 2A). Top enriched transcription factor binding site motifs within the C/EBPε peaks highlighted by CENTDIST^25^ include the expected C/EBP homodimer consensus motif ranked at the first position with a p-value ~0 (complete enriched matrices list is available in Supplementary Table 1). The *de novo* motif analysis performed by RSAT confirmed the enrichment of this motif and brought to light an interesting combination of AP-1 and C/EBP binding site motif (TGANNCAAT)^26^, suggesting that like C/EBPα^27^, C/EBPε may heterodimerize with AP-1 proteins via their leucine zipper domains (complete enriched motifs list is available in Supplementary Table 2).

We focused our attention on genes bound by C/EBPε on their promoters and differentially expressed in C/EBPε WT *vs* KO cells, which would represent target genes transcriptionally regulated by C/EBPε. We observed that 15 out of 289 repressed transcripts and 69 out of 628 activated transcripts in Gr-1int, and 25 out of 425 repressed transcripts and 177 out of 1,572 activated transcripts in Gr-1hi BM cells had a promoter binding site for C/EBPε (Supplementary data 1). This finding is consistent with our previous observations that C/EBPε works as both a transcriptional activator and a repressor of certain genes by coordinating with co-activators and co-repressors^28^. Interestingly, the 177 genes activated by C/EBPε in Gr-1hi cells are significantly enriched in gene ontology categories involved in inflammation and immune response (Supplementary Figure 2B). Moreover, our list of C/EBPε targets included lactoferrin (*Ltf*), *Camp*, neutrophil collagenase (*Mmp8*), and neutrophil gelatinase-associated lipocalin (*Ngal* or *Lcn2*) which are well-characterized C/EBPε targets, indicating the validity of our approach of combining the RNA-Seq and ChIP-Seq^15^,^16^,^23^,^24^.

### C/EBPε targets predominantly granulocytic specific genes

**Figure 1:**
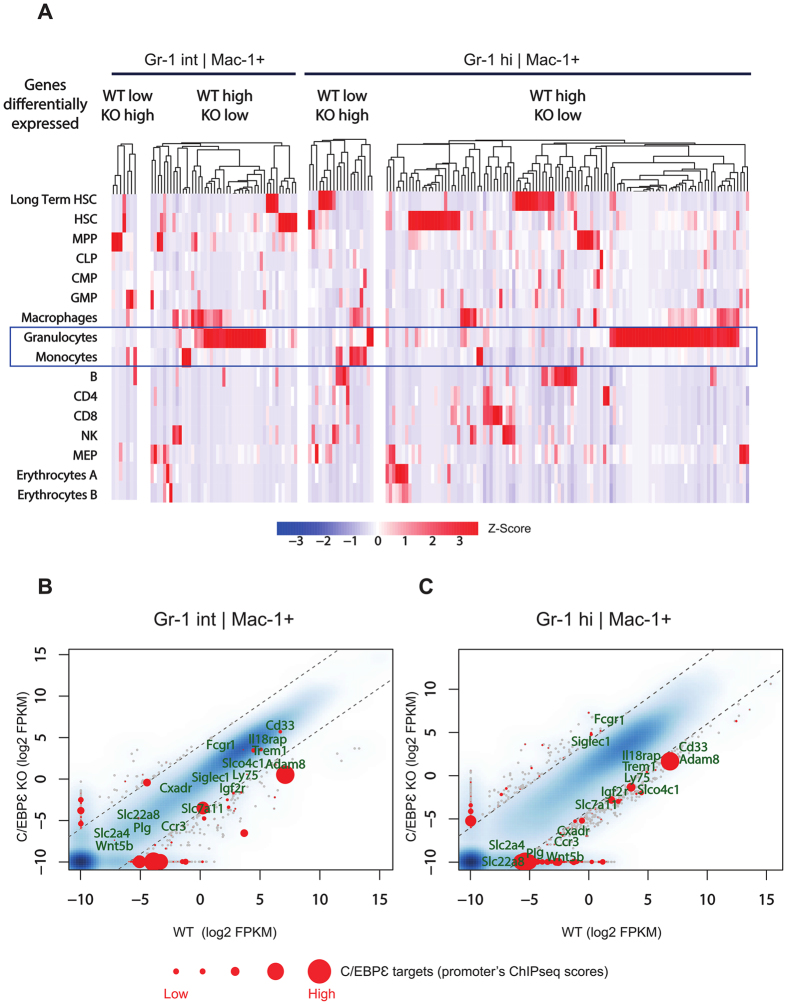
Transcriptomic signature of C/EBPε target genes. (**A**) Expression signature of genes differentially expressed in C/EBPε KO *vs* WT in both Gr-1int and Gr-1hi populations during hematopoietic differentiation. The four heatmaps depict, from left to right, the expression signature of genes either silenced (WT low, KO high) or activated (WT high, KO low) by C/EBPε in Gr-1int; and either silenced (WT low, KO high) or activated by C/EBPε (WT high, KO low) in Gr-1hi BM cells, as analyzed in different hematopoietic compartments from the RNA-Seq dataset of Lara-Astiaso *et al*. (available from the GEO omnibus database, accession number: GSE60101)^29^. (**B** and **C**) Genes differentially expressed in C/EBPε WT *vs* KO BM cells (RNA-Seq) in Gr-1int (left) and Gr-1hi (right) populations according to binary logarithm of differential expression of more than 4. Direct C/EBPε target genes (*i.*e. bound by C/EBPε within their −1000 bp/+100 bp promoter region) are depicted as red dots with a size proportional to their binding score intensity. Cell surface receptors differentially expressed in Gr-1hi cells are highlighted.

**Figure 2:**
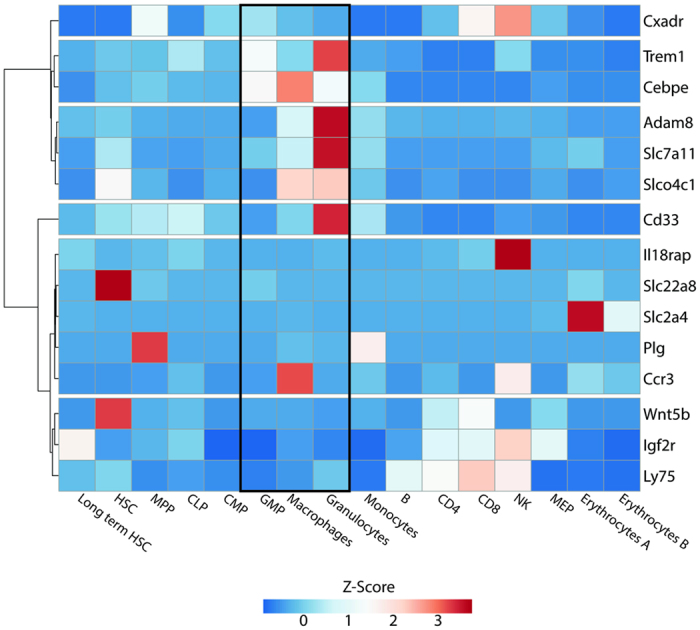
Expression profile of cell surface receptors directly regulated by C/EBPε in different hematopoietic lineages. Heatmap generated by ClustVis^49^ using Camberra distance and average linkage displays hierarchical clustered RNA-Seq expression scores from Lara-Astiaso *et al*.^29^ of fourteen cell surface receptors regulated by C/EBPε along with the transcription signature of *Cebpε*.

We analyzed the hematopoietic stage-specific expression pattern of genes which were differentially expressed in Gr-1hi and directly targeted by C/EBPε using a previously published RNA-seq data set^29^. A majority of genes activated by C/EBPε in the Gr-1int cells appear to be granulocyte-specific (Fig. 1A). Similarly, a large proportion of genes positively regulated by C/EBPε in the Gr-1hi population is specifically expressed in granulocytes (Fig. 1A), suggesting that C/EBPε is closely involved with regulation of gene network associated with terminal myeloid differentiation. These observations confirm that lack of C/EBPε impairs granulocyte development. Interestingly, 14 cell surface receptors, including five predominantly expressed on granulocytes were directly regulated by C/EBPε (Fig. 1B,C). Among them, the expression of *Trem1* was closest to the C/EBPε expression signature in different hematopoietic lineages (Fig. 2), indicating a close relationship between these two genes.

### Trem1 is a primary downstream target regulated by C/EBPε

**Figure 3:**
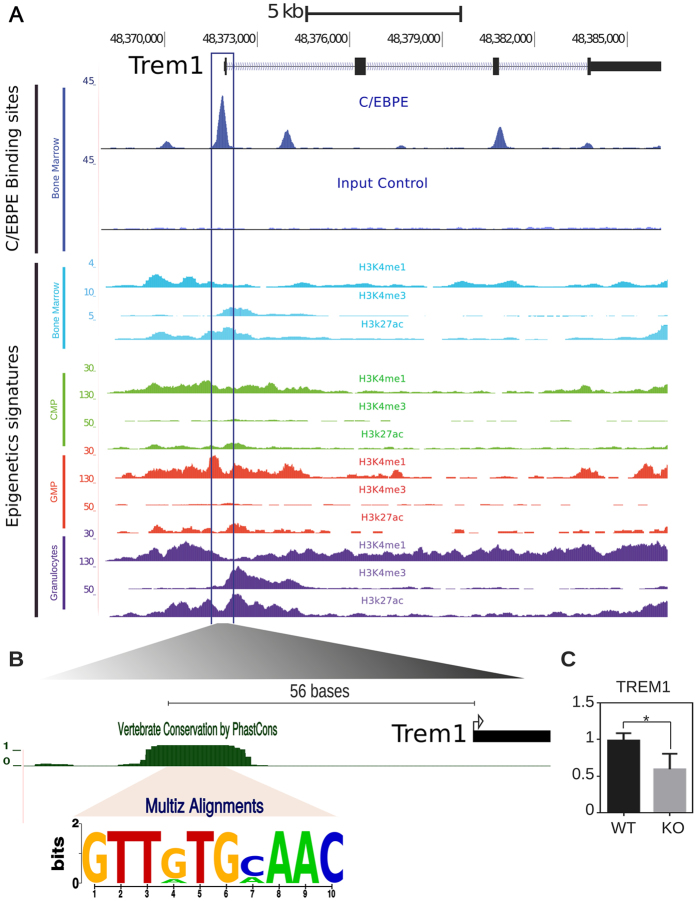
Analysis of C/EBPε binding site and epigenetic profiles within the *Trem1* promoter. (**A**) Genome browser screenshot of *Trem1* locus illustrating ChIP-Seq signal with C/EBPε antibody and input control. These results were aligned to ChIP-Seq of histone modifications, known to mark promoters (H4K4me3) and enhancers (H3K4me1, H3K27ac) from the ENCODE project^50^ (bone marrow) and Lara-Astiaso *et al*.^29^ (CMP, GMP, and granulocytes). (**B**) We characterized the precise C/EBPε binding motif by DNA sequence conservation using multi-alignment of vertebrate sequences from the UCSC *Multiz Alignment* track. Sequence logo indicates the highly conserved C/EBPε binding site consensus GTTGTGCAAC among eight species (mouse, rat, rabbit, human, chimp, orangutan, dog, and horse). (**C**) Quantitative RT-PCR was performed to measure TREM1 mRNA isolated from the Gr-1hi population of WT and KO murine BM cells (*p-value < 0.05). Relative mRNA units were normalized to glyceraldehyde-3-phosphate dehydrogenase levels.

Among 177 genes positively regulated by C/EBPε in Gr-1hi BM cells, *Trem1* was identified as one of the target genes of C/EBPε. Trem1 is a cell surface molecule expressed on differentiated neutrophils and plays a role in infectious inflammation. Trem1 stimulation is associated with a full spectrum of granulocyte functions including cell migration^17^,^18^,^30^,^31^,^32^,^33^,^34^. We identified the C/EBPε binding site within the *Trem1* promoter, in close vicinity of its TSS (−55 to −46) by ChIP-Seq and DNA sequence conservation (Fig. 3A,B). Analyses of post-translational modifications of histones within the *Trem1* promoter showed an enrichment of enhancer marks (H3Kme1, H3K27ac) in both common myeloid progenitor (CMP) and granulocyte-monocyte progenitor (GMP) populations. Both of these enhancer marks were noticeably increased in granulocytes (Fig. 3A). Moreover, the H3K4me3 signal at the Trem1 promoter was very low in CMP, increased slightly in GMP and was high in granulocytes. Altogether, these data suggest that *Trem1* transcription is primed in CMP and GMP cells and highly activated in granulocytes (Fig. 3A)^35^. Thus, the Trem1 expression coincides with C/EBPε expression in myeloid development.

**Figure 4:**
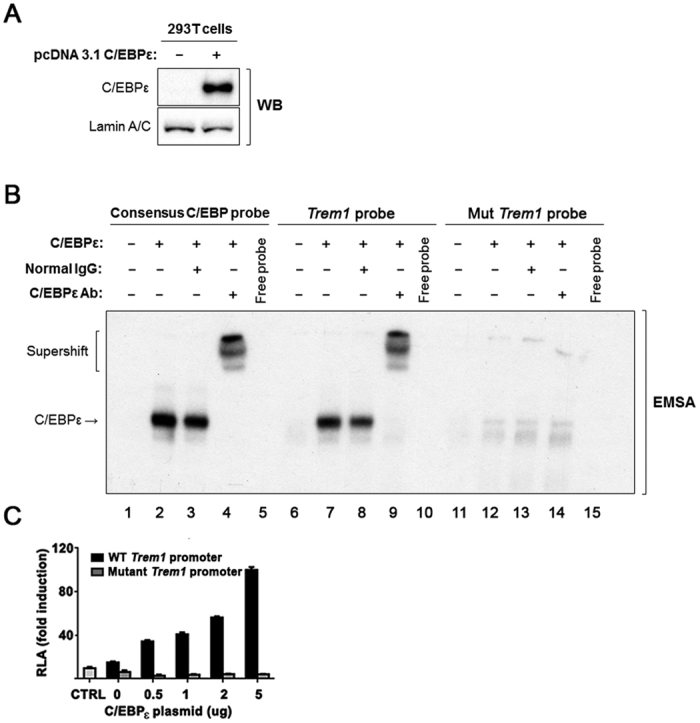
C/EBPε binds to the *Trem1* promoter and increases transcription of *Trem1*. (**A**) Western blot depicts C/EBPε protein expression in 293 T cells transfected with either empty vector or pcDNA 3.1-C/EBPε. Lamin A/C was used as loading control. (**B**) EMSA was performed with consensus C/EBP binding sequences (Consensus C/EBP probe), wild-type *Trem1* promoter sequences (*Trem1* probe), and mutant *Trem1* promoter sequences (Mut *Trem1*probe). Each radioisotope labeled probe was mixed with protein extracts from 293 T cells transfected with either a control or an expression vector for C/EBPε, and the reaction mixtures were run on native 6% polyacrylamide-TBE gel. IgG control antibody and C/EBPε antibody were added to the mixture. An arrow indicates the radioisotope labeled probe-C/EBPε complexes. Supershift indicates the radioisotope labeled probe combined with C/EBPε antibody. (**C**) pGL3 basic luciferase vectors containing either wild-type *Trem1* promoter or the promoter with a mutated C/EBP binding site were transfected into NIH3T3 cells with different amounts (0.5, 1, 2, 5 μg) of pcDNA-C/EBPε. Luciferase activities were assayed using luminometer 24 hours after transfection. Results represent fold induction of relative luciferase activity (mean ± SEM) of triplicates from two independent experiments. The relative luciferase activity of pGL3 basic is shown as a control (CTRL).

Next, we measured *Trem1* expression using quantitative RT-PCR (qPCR) in sorted Gr-1hi BM cells. We verified that the loss of C/EBPε significantly decreased the transcript levels of *Trem1* (Fig. 3C). To confirm the sequence specific interaction of C/EBPε with *Trem1* promoter, EMSA was performed with a probe containing double-stranded oligonucleotide spanning the C/EBPε binding site (GTTGTGAAAC) of the *Trem1* promoter (−69 to −36). Overexpression of C/EBPε in the 293 T cells used for EMSA was confirmed with Western blot (Fig. 4A). By mixing protein lysate from cells transfected with C/EBPε expression vector with the *Trem1* probe, a retarded band was detected (Fig. 4B, lanes 7 and 8). Supershift of this band with the addition of anti-C/EBPε antibody validated that the C/EBPε is a component of the protein complex that binds to the *Trem1* promoter (Fig. 4B, lane 9). EMSA with C/EBP consensus probe (Fig. 4B, lanes 2 to 4) and C/EBP binding site mutated *Trem1* probe (GCCAAGCCGC) (Fig. 4B, lanes 12 to 14) demonstrated the specific nature of this DNA-C/EBPε interaction. To determine the functional importance of the binding of C/EBPε to these sequences, the 253 bp upstream fragment of *Trem*1 TSS containing the C/EBPε binding site, was subcloned into a luciferase reporter vector, and the promoter activity was measured by reporter gene assays with different amount of C/EBPε in NIH3T3 cells. Luciferase activities increased in a positively correlated pattern with the amounts of C/EBPε (Fig. 4C), indicating activation of transcription by the binding of C/EBPε in the promoter sequences of *Trem1*. In summary, C/EBPε directly binds to *Trem1* promoter and regulates its transcription.

### Lower Trem1 surface expression on C/EBPε KO bone marrow granulocytes

**Figure 5:**
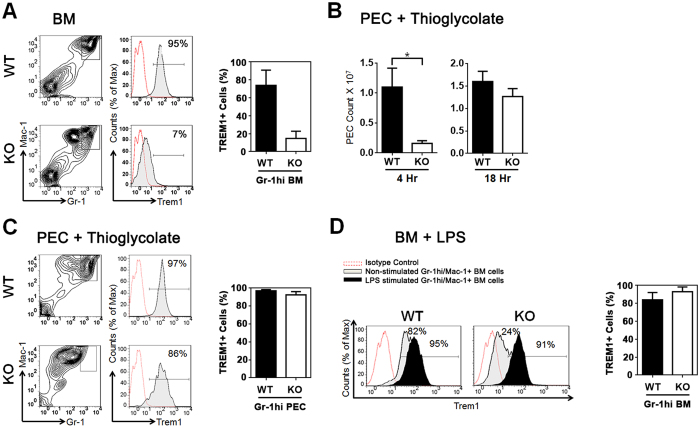
Trem1 expression in BM and PEC. (**A**) Surface expression of Trem1 on Gr-1hi granulocytes from the BM was analyzed by flow cytometry using antibodies against murine Trem1, Gr-1, and Mac-1. Contour plots and histograms show a representative staining in WT and C/EBPε KO mice. Histograms indicate Trem1 expression compared to negative control (IgG antibody; dotted line/open histogram) in Gr-1hi granulocytes (solid line/tinted histogram) in WT (upper panel) and C/EBPε KO (lower panel) from the BM. The bar graph shows a summary of Trem1 expression (%) on Gr-1hi BM cells of WT and C/EBPε KO mice (n = 6). (**B**) Number of PECs in the WT and C/EBPε KO mice were counted at 4 hours (1.1 ± 0.3 *vs* 0.16 ± 0.03 × 10^7^ cells, WT vs KO respectively, n = 6, p = 0.002) and 18 hours (1.6 ± 0.14 *vs* 1.3 ± 0.07 × 10^7^ cells, respectively, n = 6, p = 0.33) after intraperitoneal thioglycolate injection, *p < 0.01. (**C**) Trem1 expression was analyzed in PEC 18 hours after thioglycolate injection using flow cytometry. Representative FACS plots are shown. The bar graph shows a summary of the percentage of Gr-1hi PECs expressing Trem1 in WT and C/EBPε KO mice (n = 6). (**D**) Trem1 expression was examined on gated Gr-1hi granulocytes from the BM of mice 18 hours after intraperitoneal LPS injection. Histograms show Trem1 expression in WT (left panel) and C/EBPε KO (right panel) BM cells (negative control (dotted line/open histogram), unstimulated (solid line/tinted histogram) and 18 hours after LPS stimulation (solid line/filled histogram). Numbers in histograms represent the percentages of Trem1 positive Gr-1hi BM cells. The bar graph shows mean ± SEM of the percentage of Gr-1hi granulocytes expressing Trem1.

To measure protein expression of Trem1 on differentiated granulocytes, we harvested BM cells from WT and C/EBPε KO mice and performed flow cytometry. Consistent with our quantitative RT-PCR data, the proportion of Trem1 positive cells among the Gr-1hi population was significantly reduced in C/EBPε KO mice compared with the WT mice (14.8 ± 8.0% (n = 6) in KO mice compared with 73.7 ± 17.0% (n = 6) in wild type (p < 0.01) (Fig. 5A). Reduced Trem1 expression was also observed in Gr-1int BM cells of C/EBPε KO mice compared with WT (16.4 ± 9.9% *vs* 35.4 ± 27.0%, respectively, n = 6, p = 0.31).

As Trem1 has been implicated in granulocytic migration, we tested the physiologic correlation of decreased expression of Trem1 and transperitoneal migration of granulocytes *in vivo.* Thioglycolate injected intraperitoneally causes localized inflammation in the peritoneal cavity. We injected thioglycolate in the peritoneal cavity of the WT and C/EBPε KO mice, and peritoneal exudate cells (PEC) were analyzed 4 and 18 hours later. Granulocytes of C/EBPε KO mice had defective migration through the peritoneal membrane 4 hours after intraperitoneal injection of thioglycolate. By 18 hours, levels of migrating neutrophils became comparable in WT and KO peritoneal cavity (Fig. 5B). PEC at 18 hours after the thioglycolate injection were analyzed for Trem1 expression by flow cytometry demonstrating that the proportion of Gr-1hi population was significantly higher in WT PEC compared with KO PEC (35.3 ± 3.0% *vs* 3.3 ± 0.4%, respectively, n = 6), although the overall frequencies of Gr-1^+^/Mac-1^+^ cells in PEC of WT and C/EBPε KO mice were comparable (WT: 50.7 ± 5.5% *vs* KO: 72.3 ± 6.6%, n = 6, p > 0.05). This indicated that activated granulocytes of C/EBPε KO mice gained the migratory ability to the site of inflammation regardless of the extent of terminal differentiation (Fig. 5C). Interestingly, while BM Gr-1hi cells of C/EBPε KO mice consistently showed lower level of Trem1 expression even in the thioglycolate treated mice (84.8 ± 5.0% *vs* 33.9 ± 5.8, WT *vs* KO, respectively, n = 6, p < 0.01), Trem1 expression in PEC Gr-1hi cells of C/EBPε KO mice was as high as the wild type counterpart (98 ± 7.2% *vs* 92.0 ± 7.1, WT *vs* KO, respectively, n = 6, p > 0.05) (Fig. 5C). These data indicate that inflammatory signals by thioglycolate up-regulate the expression of Trem1 and demonstrate differential Trem1 expression in PEC from non-primed C/EBPε KO BM cells.

### LPS stimulation increases Trem1 expression on differentiated granulocytes of C/EBPε KO mice

Cross-linking of the surface TLR4 with its specific ligand, lipopolysaccharide (LPS), results in activation of granulocytes and increased Trem1 expression and cytokine production via NF-*k*B signal transduction^36^,^37^. Therefore, we next determined whether the decreased Trem1 expression is compensated by LPS stimulation in C/EBPε KO BM granulocytes.

**Figure 6:**
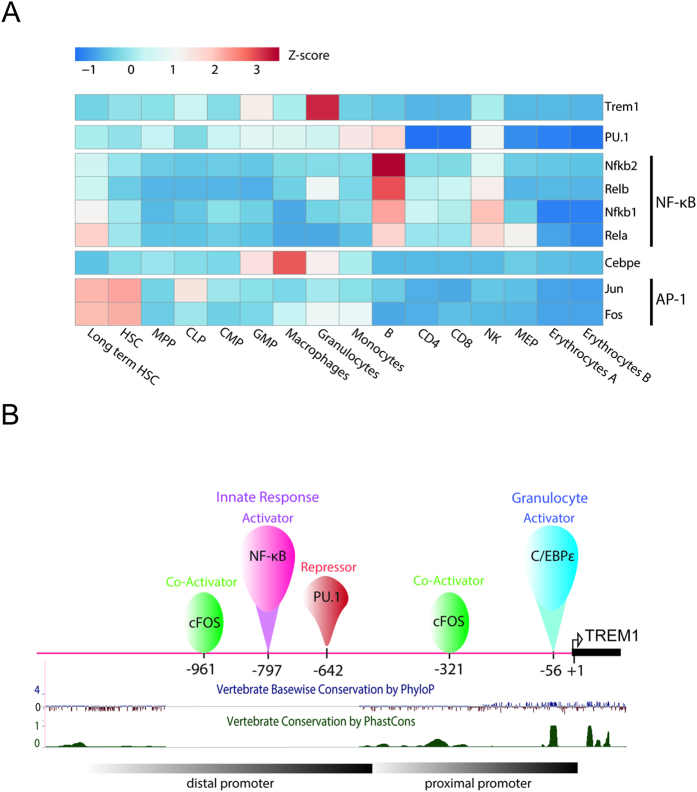
Analysis of transcription factors binding to *Trem1* promoter. (**A**) RNA-Seq expression profile analysis of characterized *Trem1* regulators in hematopoietic lineage^29^ shows that C/EBPε expression signature is similar to *Trem1* compared to other transcription factors. (**B**) Model of *Trem1* dual activation mode. Two regulatory regions (distal and proximal promoters) regulate *Trem1* activation. The distal region hosts an NF-*k*B binding site involved in *Trem1* activation induced by an inflammatory response; while the proximal promoter contains a C/EBPε binding site which regulates the basal *Trem1* transcription in granulocytes. Notably, the USCS tracks “Vertebrate Basewise Conservation by PhyloP” and “Vertebrate Conservation by PhastCons” show that C/EBPε binding site is much more conserved among vertebrates than the NF-*k*B binding site.

LPS that induces systemic inflammation was intraperitoneally injected into WT and C/EBPε KO mice, and the Trem1 expression on Gr-1hi granulocytes in the BM was measured at 18 hours after injection. Flow cytometry of C/EBPε KO Gr-1hi BM cells showed that Trem1 expression in Gr-1hi BM cells was higher than those in the non-stimulated condition, becoming comparable to WT Gr-1hi BM cells (83.7 ± 3.3% *vs* 92.8 ± 2.1% respectively, p > 0.05) (Fig. 5D). These findings suggest that C/EBPε regulates the basal level of Trem1 during differentiation of granulocytes, and other signal transduction pathways or transcription factors induce Trem1 expression upon inflammatory stimulation. With LPS stimulation, NF-*k*B rapidly binds to target DNA sequences^38^. Furthermore, NF-*k*B binding sites were characterized in the *Trem1* distal promoter at −793 from its TSS^19^. Therefore, after inflammatory stimulation, NF-*k*B most likely is the transcription factor stimulating Trem1 expression in C/EBPε KO granulocytes (Fig. 6A,B).

In summary, these results demonstrate that C/EBPε independently regulates the basal Trem1 expression during terminal differentiation of granulocytes, while LPS activates Trem1 expression as an inflammatory response.

## Discussion

Neutrophils are important cells in innate immunity involved in migration to inflammation, phagocytosis, release of granules and formation of neutrophil extracellular traps. However, the molecular mechanisms regulating the cellular immune function of granulocytes are poorly understood.

C/EBPε is a critical transcription factor that mediates normal granulocyte function. To gain a full understanding of the global regulatory networks controlled by C/EBPε, we combined gene expression profile of WT and C/EBPε KO granulocytes (both Gr-1hi and Gr-1int BM cells) with genome-wide C/EBPε binding sites characterized by ChIP-Seq in bone marrow cells. We identified 40,517 C/EBPε binding sites throughout the genome. Ten percent of these binding sites were located in promoter regions (−1000 bp/+100 bp from the TSS). Our ChIP-Seq/transcriptomic integrative analysis highlighted 202 transcripts, including *Trem1*, which are bound by C/EBPε and are significantly dysregulated (expression fold change greater than 4) in C/EBPε KO mice during the terminal differentiation of granulocytes.

Trem1 is expressed on differentiated granulocytes^17^. It can activate the full spectrum of effector functions of granulocytes including the release of inflammatory cytokines, degranulation, phagocytosis, and the oxidative burst^18^,^39^,^40^. Trem1/3 double KO mice displayed a high mortality rate by 24 hours after Gram-negative bacterial challenge secondary to dysregulation of cytokine production, and a migration defect of granulocytes through respiratory epithelial cells^41^, which is similar to C/EBPε KO mice^16^. The finding that both C/EBPε KO mice and Trem1/3 double KO mice exhibit increased susceptibility to infection indicates their overlapping function towards innate immunity mediated by granulocytes. We demonstrated that C/EBPε binds to the *Trem1* promoter sequences and regulates its expression. We observed a correlation of Trem1 protein expression and defect of granulocytes migration in C/EBPε KO mice. As an effector function of granulocytes, we analyzed their migration through the peritoneal membrane after thioglycolate injection. Consistent with a previous report^14^, we observed a significantly fewer number of PEC in C/EBPε KO mice in the early phase (4 hours) after injection, which parallels decreased level of Trem1 on Gr-1hi granulocytes. But the comparable number of PEC was obtained in a later phase (18 hours) with the restored level of Trem1 in Gr-1hi granulocytes of C/EBPε KO mice. The Gr-1hi PEC at 18 hours after thioglycolate injection showed higher Trem1 expression than the same Gr-1hi granulocytes in BM, which suggests that inflammatory signals in the peritoneal cavity induced Trem1 expression and successfully recruited granulocytes of C/EBPε KO mice across the peritoneal membrane. On systemic LPS challenge that activates NF-*k*B pathway through Tlr4, Trem1 expression in C/EBPε KO Gr-1hi granulocytes in BM was similar to the levels present in WT Gr-1hi granulocytes in BM. Therefore, we could conclude that inflammatory signals compensated for a lower level of Trem1 expression secondary to C/EBPε deletion, and rescued the migratory defect of Gr-1hi granulocytes in C/EBPε KO mice. This inspection was supported by finding a NF-*k*B binding site on the distal *Trem1* promoter area (−797 bp)^19^. Finally, these data suggest that enforced expression of Trem1 might rescue decreased innate immunity of patients who have dysfunctional C/EBPε.

Though C/EBPε does not share a DNA-binding site on the *Trem1* promoter with NF-*k*B, our previous study showed that C/EBPε interacted with p38 MAP kinase and p65RelA. Once p38 MAP kinase phosphorylates C/EBPε at Thr75, its DNA binding capacity is highly up-regulated by interaction with the activated NF-*k*B pathway protein p65RelA^42^,^43^. Therefore, we speculate that in mature WT granulocytes, the Trem1 expression is potentiated upon inflammation by C/EBPε in collaboration with NF-*k*B and p38 MAP kinase. By regulating *Trem1* transcription in steady state granulopoiesis, C/EBPε prepares granulocytes to respond to infection. Furthermore, C/EBPε also enhances Trem1 expression via LPS induced inflammatory signal on differentiated granulocytes in inflammation.

In conclusion, by utilizing ChIP-Seq and RNA-Seq, this study demonstrates direct target genes of C/EBPε and provides a comprehensive landscape of the role of C/EBPε in terminally differentiated granulocytes. We identify Trem1 as a novel downstream effector of C/EBPε function in innate immunity. We anticipate that further analysis of C/EBPε target genes will add more insights into its role in granulocytic differentiation and function, which may pave the way for developing new immunotherapeutic approaches.

## Methods

### Mice

The C/EBPε KO mice provided by Drs K.G. Xanthopoulos and Julie Lekstrom Himes were on the 129/SvEv strain^7^. The C/EBPε KO mice were backcrossed onto a pure C57BL/6 background (more than eight generations) and were bred and maintained at Burns and Allen Research Institute at Cedar-Sinai Medical Center. The loss of C/EBPε was verified by genotyping (primers: forward: GCTACAATCCCCTGCAGTCC, reverse for WT: TGCCTTCTGCCCTTGTG, for KO: ATCGCCTTCTATCGCCTTCTTGACGAG). All mice were maintained under specific pathogen-free conditions. Animal care and use were in accordance with the procedures outlined in the Guide for the Care and Use of Laboratory Animals under an animal study proposal approved by the Cedars-Sinai Medical Center Institutional Animal Care and Use Committee.

### Bone marrow cell preparation and flow cytometry

Bone marrow was flushed from femurs and tibias of the mice. Red cells were lysed using ACK buffer. Following incubation in IMDM medium for 10 minutes, BM cells were washed and suspended in phosphate-buffered saline (PBS). The cells were stained with antibodies to Gr-1(FITC), Mac-1 (PE) and Trem1 (APC). Antibodies for flow cytometry were purchased from eBioscience (San Diego, CA) and R&D systems (Minneapolis, MN). Cell-antibody mixtures were kept at 4 °C for 30 minutes followed by two washes with PBS/1%FBS. Flow cytometry was performed on Cyan ADP Analyzer (Beckman Coulter, Brea, CA) and analyzed using FlowJo software (Tree Star Inc, Ashland, OR).

### RNA-Sequencing

Gr-1hi (Gr-1high/Mac-1^+^) and Gr-1int (Gr-1intermediate/Mac-1^+^) granulocytes were sorted from bone marrow of CEBPε KO and WT mice using FACS ARIA (BD Biosciences). Sorted cells were lysed, and RNA was extracted using Qiagen RNeasy Micro kit. cDNA libraries were prepared using TruSeq RNA Sample Preparation Kit (Illumina) according to the manufacturer’s protocol and sequenced on HiSeq 4000 (Illumina).

Sequenced reads were aligned to the mouse reference genome mm9 using STAR with default parameters^44^. FPKM (fragments per kilobase of exon per million) scores were generated using the “analyzeRepeats.pl” script from the Homer package^45^, and transcript log fold-changes were computed in R (https://www.r-project.org). Differential transcript expression was defined as the binary logarithm of differential expression more than 4 in Gr-1hi or Gr-1int cells between WT and C/EBPε KO (log_2_WT/KO ratio ≥ 4).

### Data integration

Our datasets were integrated with publicly available RNA-Seq generated by Lara-Astiaso *et al*.^29^ from the GEO omnibus database, accession number: GSE60101. Z-Score expression scores were computed from the RNA-Seq expression quantification using the pheatmap library from R/Bioconductor (http://cran.r-project.org/web/packages/pheatmap/index.html). Cell surface proteins were identified using the Surfaceome database (http://www.imm.ox.ac.uk/surfaceome-database). In our analysis, we selected only membrane receptors under the class “gold”.

### Chromatin immunoprecipitation (ChIP) and sequencing

BM cells were harvested from femurs and tibias of C57BL/6 mice. After red cell lysis, cells were cross-linked in 1% formaldehyde. Cells were lysed, and chromatin was sheared in SDS lysis buffer (containing 1% SDS) using Bioruptor sonicator (Diagenode) (40 cycles of 1 min each; 30 sec on, 30 sec off). Lysates were precleared with Dynabeads Protein A+ Dynabeads protein G (Life Technologies, Carlsbad, CA) for 90 min at 4 °C. Immunoprecipitation was performed with the anti-CEBPε antibody (GeneTex, GTX109155) bound to Protein A/G Dynabeads for overnight at 4 °C. Immunocomplexes were washed and eluted from beads in 1% SDS, 0.1 M sodium bicarbonate, followed by reverse crosslinking at 65 °C for 12–14 hours. DNA was purified using Qiagen QIAquick PCR Purification Kit. ChIP-DNA was quantified using Qubit dsDNA High-Sensitivity Assay Kit (Life Technologies) and enrichment of specific chromatin fragments was verified by quantitative PCR. Adapter sequences were ligated to DNA fragments, followed by PCR amplification and size selection (100–300 bp). Libraries of ChIP-DNA were sequenced on HiSeq 4000 (Illumina).

ChIP-Seq reads were mapped to the mouse reference genome build 37 (mm9) using Bowtie2 with default parameters^46^. ChIP-Seq signals were visualized using the UCSC Genome Browser (http://genome.ucsc.edu). ChIP-Seq peaks calling and annotation were performed using the modules “findPeaks” (default parameters) and “annotatePeaks.pl” respectively, from the Homer package^45^. Input DNA sequencing was used as a background baseline for identifying significant ChIP-Seq peaks. Transcription factor binding site-matrices from Jaspar were scanned throughout all peaks using CENTDIST with default parameters^25^. Overrepresented DNA motifs within C/EBPε binding sites were characterized using the “peak-motifs” module from the RSAT web server using the following parameters: -max seq len 1000 –markov auto –disco oligos,dyads,positions,local words –nmotifs 10 –minol 6 –maxol 8 –merge lengths -2str –origin center –motif db jaspar core nonredundant vertebrates^26^.

### Isolation of mouse peritoneal granulocytes

Thioglycolate-induced peritoneal neutrophils were prepared as described in a previous article^47^. Briefly, 2 ml of 4% thioglycolate broth (Sigma Chemical Co., St. Louis, MO) was intraperitoneally injected. After 4 and 18 hours of injection, the peritoneal lavage was collected with cold phosphate buffered saline containing 0.2% albumin.

### Reverse transcription and quantitative PCR

Total RNA was extracted using the RNeasy kit (Qiagen, Valencia, CA) and cDNA was prepared from 1 µg total RNA using Retroscript High Capacity cDNA Reverse Transcription kit (Life Technologies) according to manufacturer’s instructions. Quantitative PCR was performed using a CFX96 qPCR system and SYBR Green supermix. Expression levels of glyceraldehyde-3-phosphate dehydrogenase (GAPDH) were used for normalization. Primers used for *Trem1* are Forward: 5′-TCCACCTCCTGTGAAGATCC-3′, Reverse: 5′-AAAGACGTGTCCCAGAGCAC-3′.

### EMSA

293T cells were transfected with either 1 μg pcDNA3.1(−) empty vector or C/EBPε expressing vector in 100 mm dishes using X-tremeGENE HP (Roche) according to manufacturer’s instructions. After transfection, cells were cultured for 48 hours before harvesting. Nuclear extracts were prepared from transfected 293T cells, as described^48^. The following antibodies were used: C/EBPε (sc-158; Santa Cruz); Lamin A/C (#2032; Cell Signaling); and anti-rabbit horseradish peroxidase-conjugated antibody (W4018; Promega). EMSA was performed as described^48^. Briefly, double-stranded oligonucleotide probes were labeled with [γ-^32^P] ATP using T4 polynucleotide kinase (Roche). The following probes were used (C/EBP binding sites are underlined): C/EBP consensus (5′-GATCCATATCCCTGATTGCGCAATAGGC TCAAAA); *Trem1* (5′-TGGCCTCACATCCTGTTGTGAAACTTTCCAGAGACTAG); Mutant *Trem1* (5′-TGGCCTCACATCCTG***CCAA***G***CCG***CTTTCCAGAGACTAG). Labeled probes were incubated with nuclear extracts in 20 mM HEPES, pH 7.5, 200 mM NaCl, 5% Ficoll, 5 mM DTT, 5 mM EDTA, 40 ng/μL of poly-d(I-C), and 40 ng/μL of BSA at room temperature for 20 min. For supershift assays, nuclear extracts were pre-incubated with 200 ng of normal rabbit IgG (sc-2027; Santa Cruz) or anti-C/EBPε (sc-158; Santa Cruz) for 20 min at room temperature prior to the binding reactions. DNA-protein complexes were resolved on native 6% polyacrylamide–TBE gels.

### Luciferase assay

The 253 bp upstream fragment of *Trem*1 TSS containing the C/EBPε binding site was amplified by PCR using mouse bone marrow mononuclear cells as a template and subcloned into a pGL3-Basic vector (Promega, Madison, WI). The sequence was verified using an ABI PRISM 3100/3130 Genetic Analyzer (Life Technologies). NIH3T3 cells (6 × 10^5^ cells/well in 2 ml DMEM media) were transfected with pcDNA-C/EBPε and the pGL3 basic vector containing wild-type *Trem1* promoter sites (TTGTGAAA) or mutated C/EBP binding sites (CCAAGCCG) using Lipofectamine Plus (Life Technologies). Twenty-four hours after transfection, luciferase activities were measured using Promega Dual-Glo assay kit. The Renilla basic vector was cotransfected as a control for normalization.

### Statistics

Statistical analysis was performed using two-tailed Student’s t-test using Prism 6 software (GraphPad, La Jolla, CA). Data from each experiment are presented as mean values ± SEM. Differences of p < 0.05 were considered significant.

### Accession numbers

RNA-Seq and ChIP-Seq datasets are available at GEO (www.ncbi.nlm.nih.gov/geo) under the accession number GSE73860.

## Additional Information

**How to cite this article:** Suh, H. C. *et al*. LPS independent activation of the pro-inflammatory receptor Trem1 by C/EBPε in granulocytes. *Sci. Rep.* **7**, 46440; doi: 10.1038/srep46440 (2017).

**Publisher's note:** Springer Nature remains neutral with regard to jurisdictional claims in published maps and institutional affiliations.

## Supplementary Material

Supplementary Figures
