# Supplementary material for: LPS independent activation of the pro-inflammatory receptor Trem1 by C/EBPε in granulocytes
Source: Sci Rep. 2017 Apr 25;7:46440. doi: 10.1038/srep46440 (PMC5404328; doi:10.1038/srep46440)
Supplement: Supplementary Figures [file srep46440-s1.doc]

Supplementary information of

**LPS independent activation of the pro-inflammatory receptor Trem1 by C/EBPε in granulocytes**

Hyung C. Suh^1,#^,*, Touati Benoukraf^2,#^,*, Pavithra Shyamsunder^2,#^, Tong Yin^3,#^, Qi Cao^3^, Jonathan Said^4^, Stephen Lee^4^, Ricky Lim^2^, Henry Yang^2^, Jacqueline Salotti^5^, Peter F. Johnson^5^, Vikas Madan^2^,*^,§^ H. Phillip Koeffler^2,3,6,§^

**Supplementary Figures:**


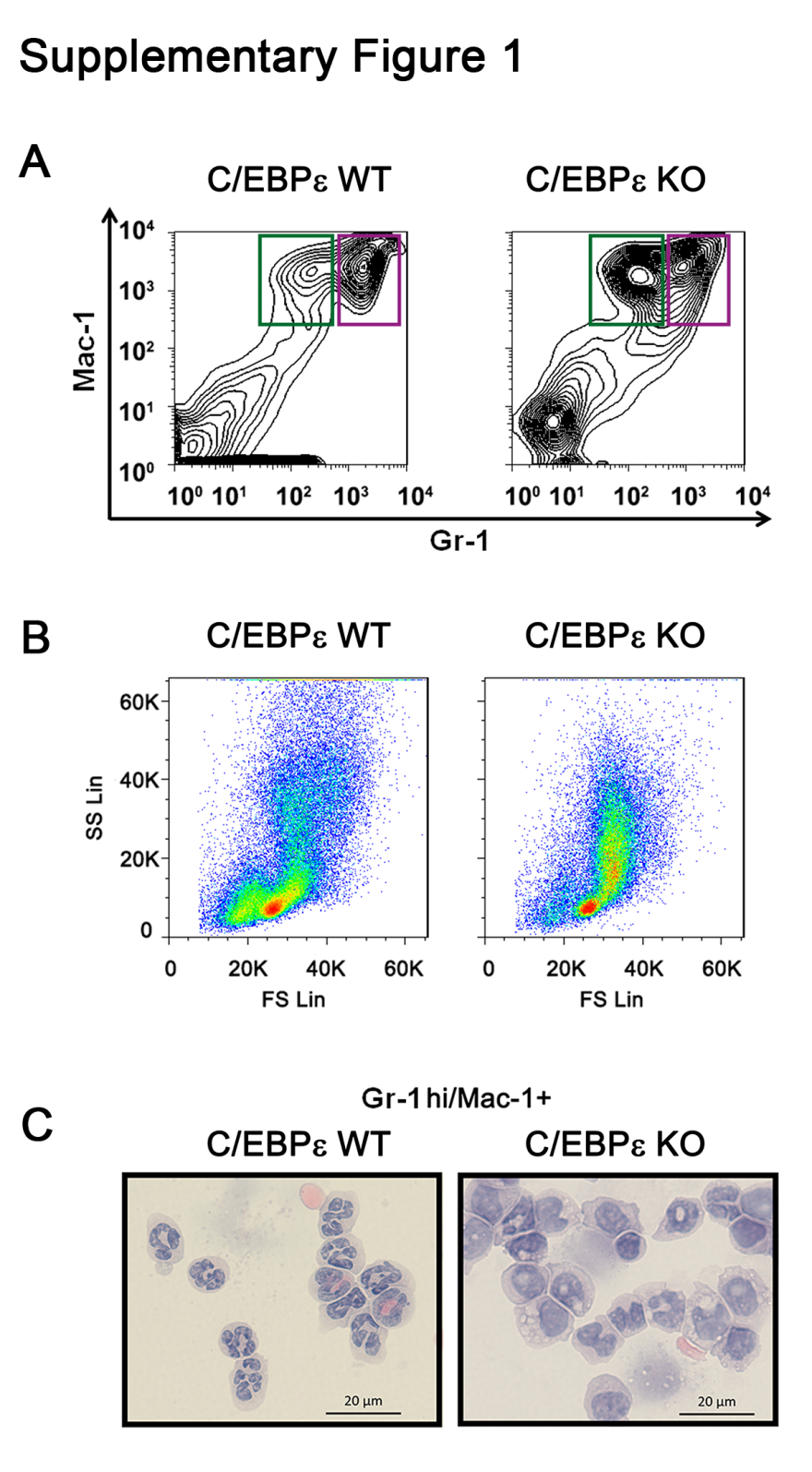


**Supplementary Figure 1.** **Morphologic differences in granulocytic cells of wild type and C/EBPε KO mice.** (A) Representative flow cytometry plots showing myeloid BM cells in WT and KO mice. Two gated populations depict Gr-1intermediate/Mac-1+ (Gr-1int) and Gr-1high/Mac-1+ (Gr-1hi) cells. (B) Forward scatter (FS) versus side scatter (SS) plots of whole bone marrow cells from wild-type and C/EBPε KO mice. The dot plot of KO mouse bone marrow demonstrates less granularity (lower SS). (C) Representative morphology of Gr-1high/Mac-1+ cells from PEC 18 hours after intraperitoneal injection of thioglycolate. Images shown are either from wild-type (WT) or C/EBPε KO mice; the latter as an example of abnormal granulocyte morphology. Compared with H&E-stained wild-type cells, which are dominated by granulocytes with thin, segmented nucleus, KO cells demonstrate poorly segmented, thick nucleus. All images were produced at room temperature, using a Zeiss ImagerM2 microscope and an Axiocam 506 mono (Carl Zeiss Microscopy GmbH). Zeiss Zen imaging software; ZEN2 blue edition (www.zeiss.com/microscopy) was used to capture the images. Magnification: ×630. Scale bars, 20 μm.


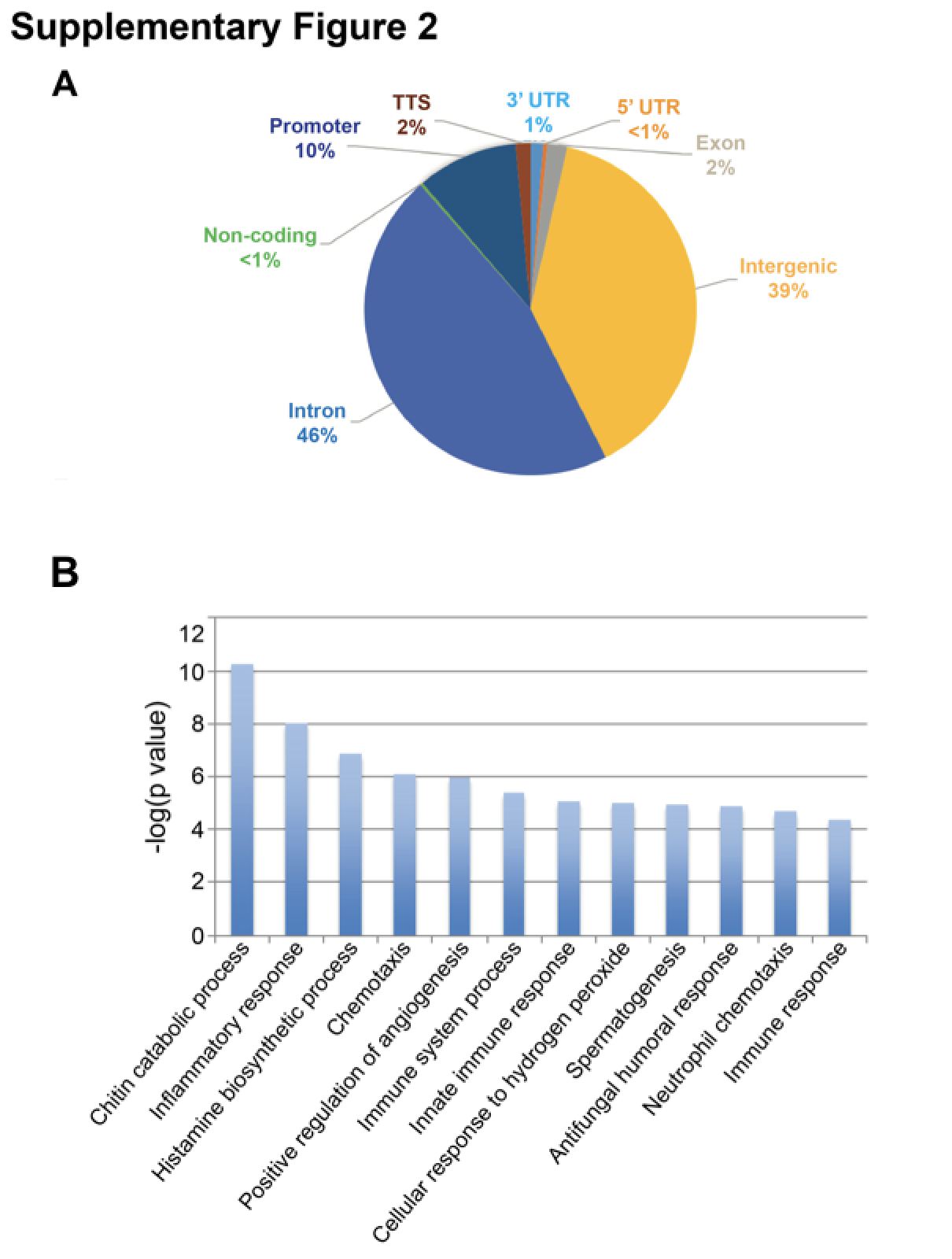


**Supplementary Figure 2. Characterization of C/EBPε ChIP-Seq peaks. (**A) Genomic annotation of the 40,517 C/EBPε binding sites according to known RefSeq genes. Promoter-associated sites are defined as the regions ranging from -1000 to +100bp from the transcription start sites. (B) Gene Ontology (Molecular Function) enrichment of genes differentially expressed in Gr-1hi in our RNA-Seq analysis and directly targeted by C/EBPε.

**Supplementary Tables**


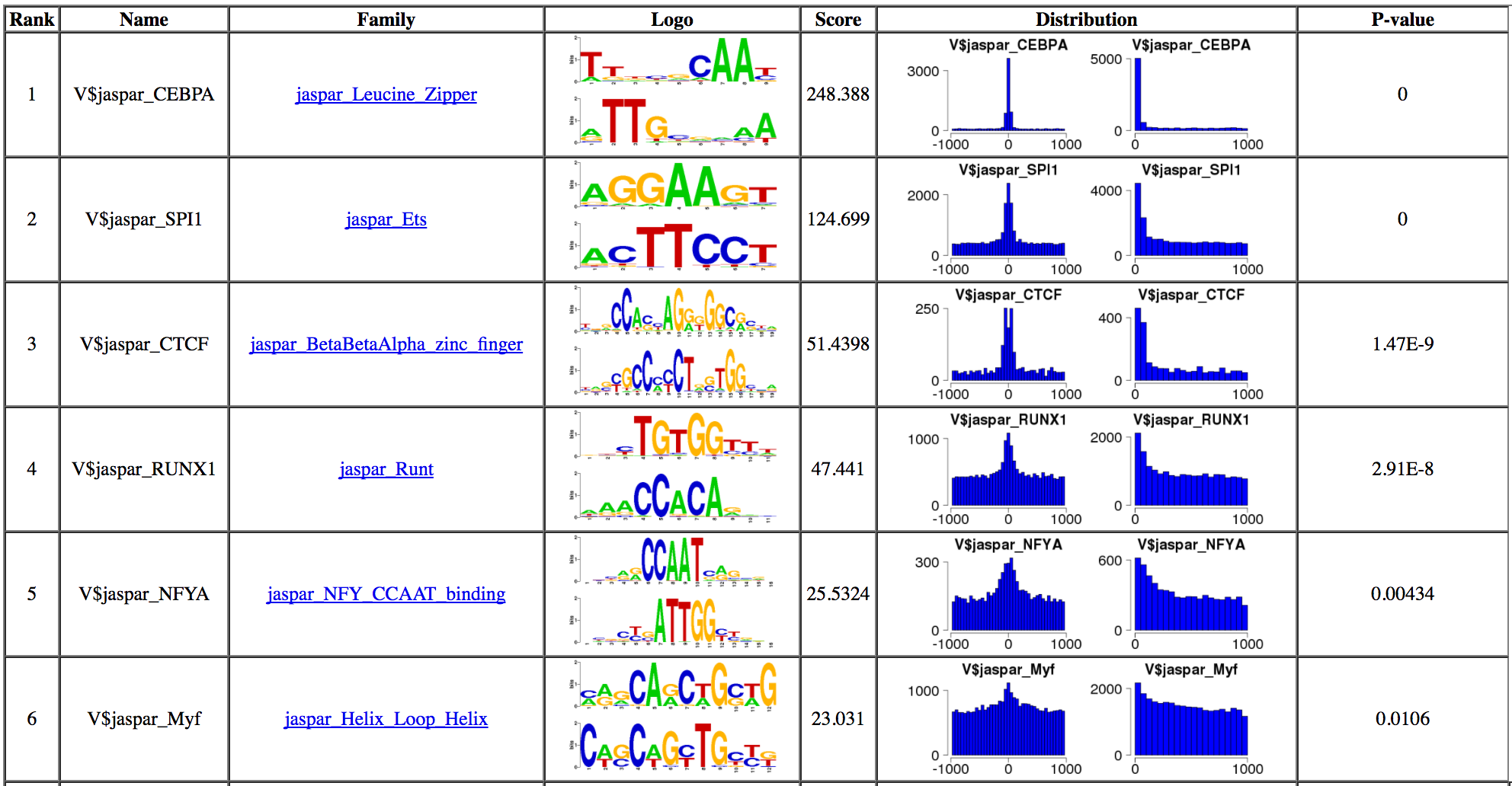


**Supplementary Table 1. List of enriched Jaspar matrices within C/EBPε** **binding sites.** Analysis of enriched Jaspar matrices performed by CENTDIST using default parameters, across C/EBPε binding sites shows that C/EBPε binds the expected homodimer motif ATTGNNCAAT (Rank #1). Interestingly, C/EBPε binding sites seem to co-localize with crucial hematopoietic transcription factors binding sites such as PU1 (SPI1, Rank #2) and RUNX1 (Rank #4).

| **Motif Algorithm ID** | **Logo** | **3 Top hits in databases  (**jaspar Core Vertebrates**)** | **E-Value** |
| --- | --- | --- | --- |
| positions_6-8nt_m1 | 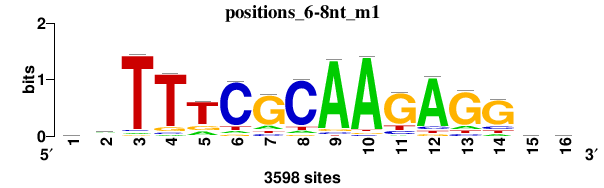 | CEBPB, CEBPE, CEBPD | 1e-300 |
| positions_6-8nt_m2 ****** | 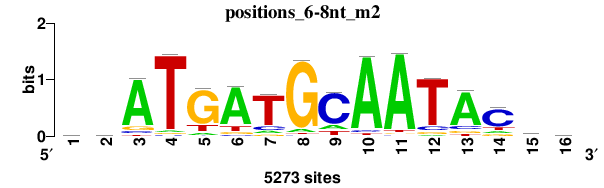 | ATF4, CEBPA, JUN **Potential CEBPE/AP1 heterodimer** | 1e-300 |
| positions_6-8nt_m3 | 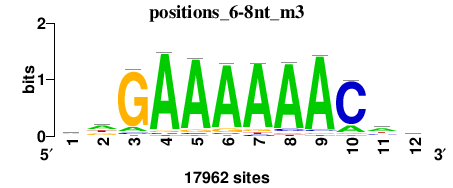 | NFATC2 | 1e-300 |
| positions_6-8nt_m4 | 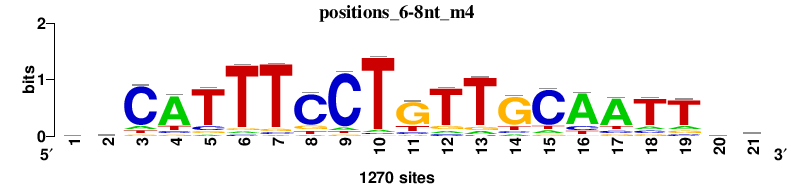 | ETV2 | 1e-300 |
| positions_6-8nt_m5 | 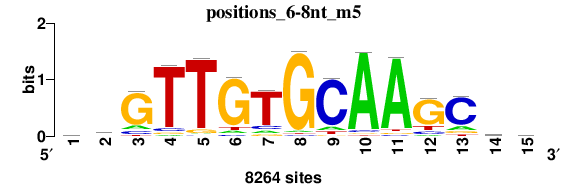 | CEBPA, CEBPE, CEBPB | 1e-300 |
| positions_6-8nt_m6 | 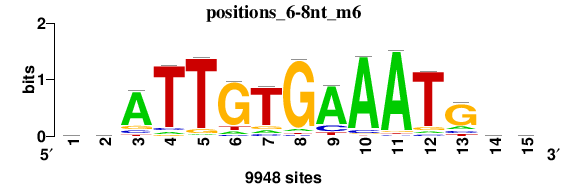 | CEBPA, CEBPE, Sox17 | 1e-300 |
| positions_6-8nt_m7 | 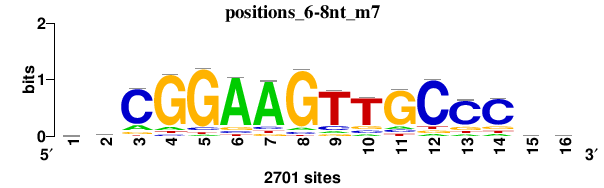 | ETV6, ELK3, ELF5 | 1e-300 |
| positions_6-8nt_m8 | 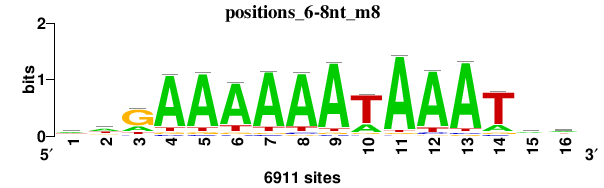 | ONECUT3, Foxd3 | 1e-300 |
| positions_6-8nt_m9 | 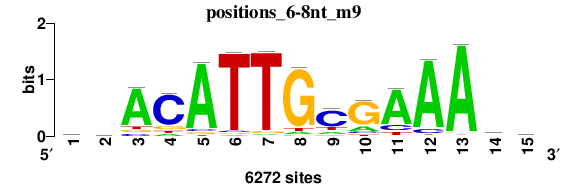 | CEBPE, CEBPD, CEBPA | 1e-300 |
| positions_6-8nt_m10 | 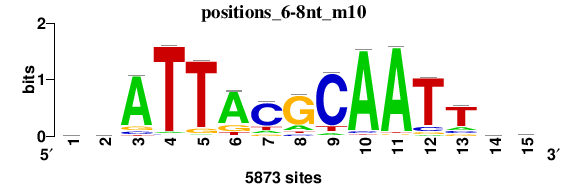 | CEBPD, CEBPB, CEBPG | 1e-300 |
| local_words_6-8nt_m1 | 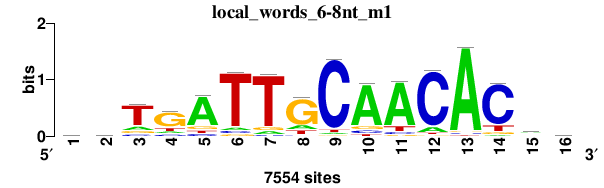 | CEBPA, Dux | 0 |
| local_words_6-8nt_m2 ***** | 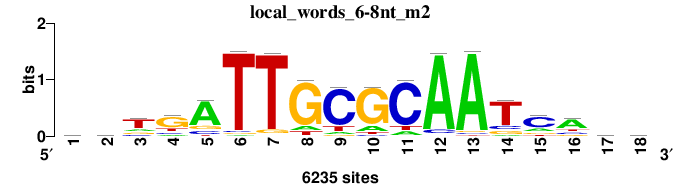 | CEBPG, CEBPD, CEBPE  **CEBP Homodimer motif** | 0 |
| local_words_6-8nt_m3 | 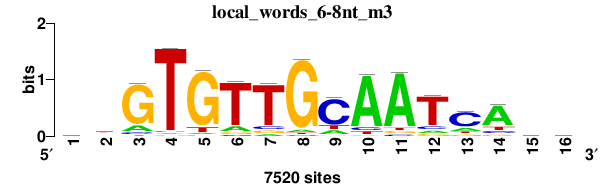 | Dux, CEBPA | 0 |
| local_words_6-8nt_m4 | 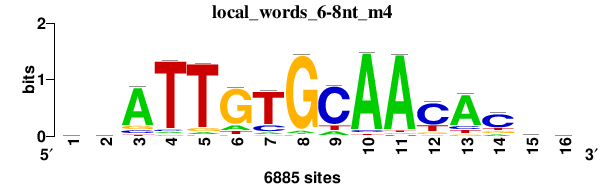 | CEBPA, CEBPE, CEBPB | 0 |
| local_words_6-8nt_m5 | 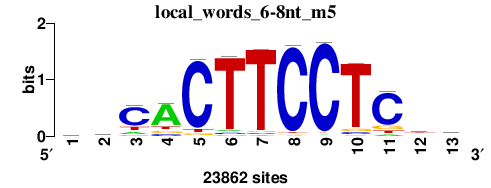 | ETV6, SPIB, ELF5 | 0 |
| local_words_6-8nt_m6 ***** | 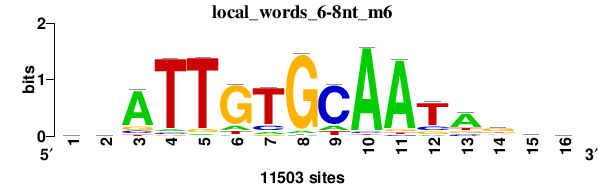 | CEBPA, CEBPE, CEBPG  **CEBP Homodimer motif** | 0 |
| local_words_6-8nt_m7 ****** | 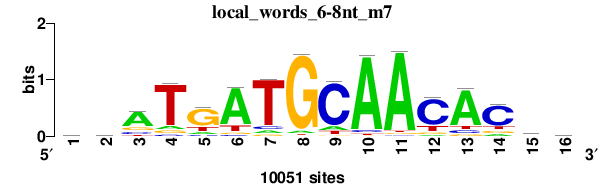 | ATF4, CEBPA  **Potential CEBPE/AP1 heterodimer** | 0 |
| local_words_6-8nt_m8 | 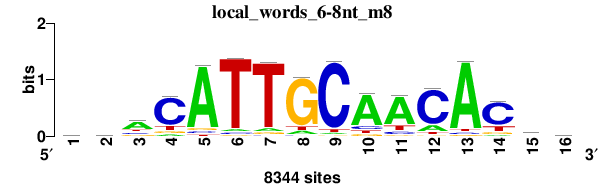 | CEBPA | 0 |
| local_words_6-8nt_m9 | 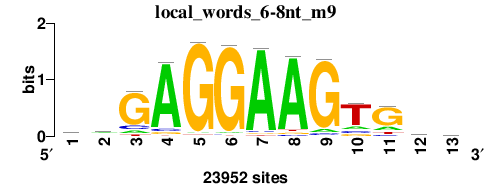 | ETV6, ELF5, SPIB | 0 |
| local_words_6-8nt_m10 ***** | 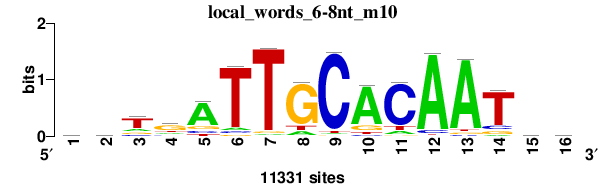 | CEBPA, CEBPE, CEBPG  **CEBP Homodimer motif** | 0 |
| oligos_6-8nt_m1 | 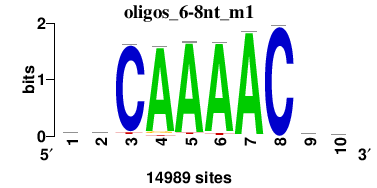 | Sox5 | 1.6e-123 |
| oligos_6-8nt_m2 | 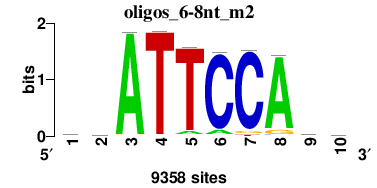 | TEAD4, TEAD1, NFATC1 | 2.4e-120 |
| oligos_6-8nt_m3 | 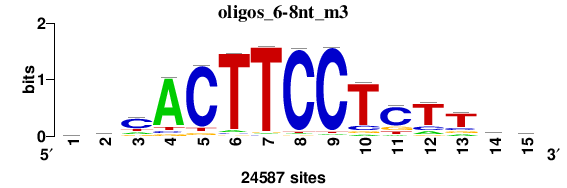 | SPIC, ETV6, SPIB | 2.7e-116 |
| oligos_6-8nt_m4 | 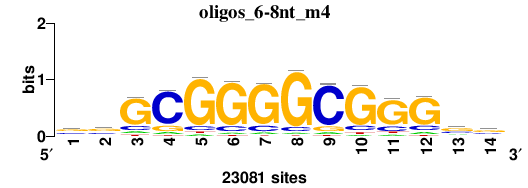 | SP2, KLF5, SP1 | 1.8e-98 |
| oligos_6-8nt_m5 | 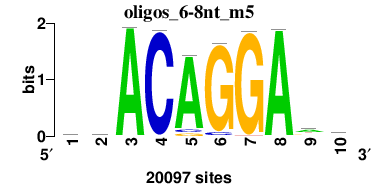 | ETS1, ETV2, ERF, | 3.6e-81 |
| oligos_6-8nt_m6 | 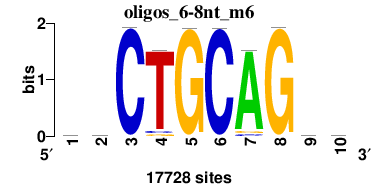 | no match | 4.5e-75 |
| oligos_6-8nt_m7 | 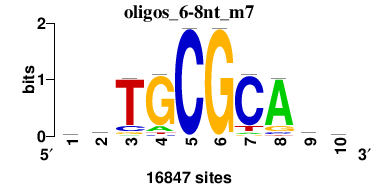 | CEBPG, NRF1, CEBPB | 2.9e-69 |
| oligos_6-8nt_m8 | 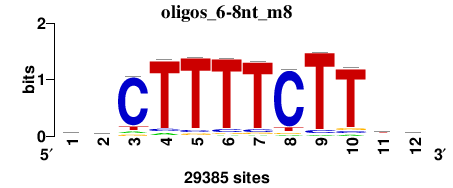 | GATA3 | 4e-68 |
| oligos_6-8nt_m9 | 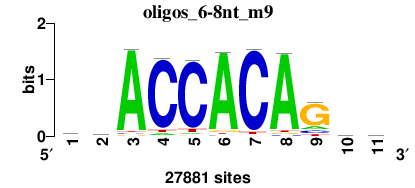 | RUNX1, RUNX3, RUNX2 | 1.5e-66 |
| oligos_6-8nt_m10 | 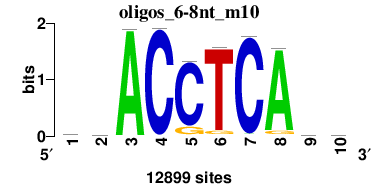 | CREB1, RUNX2, Crem | 1.7e-59 |
| dyads_m1 | 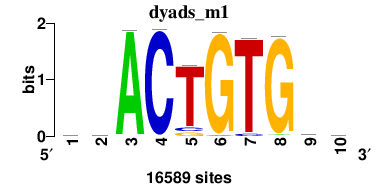 | no match | 0 |
| dyads_m2 | 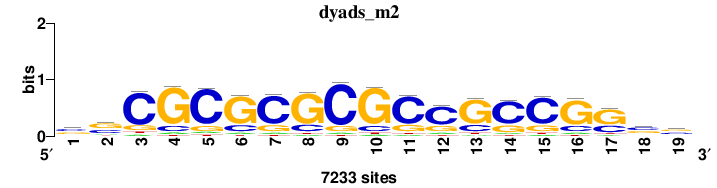 | NRF1 | 0 |
| dyads_m3 | 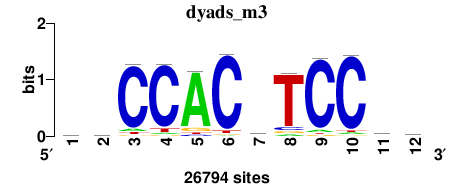 | ZNF354C, ETV6, ETV3 | 0 |
| dyads_m4 | 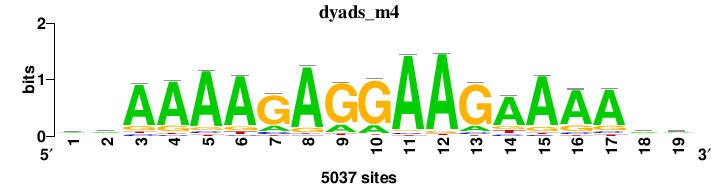 | SPIC, SPI1 | 0 |
| dyads_m5 | 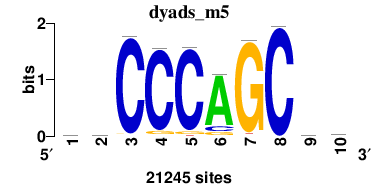 | no match | 0 |
| dyads_m6 | 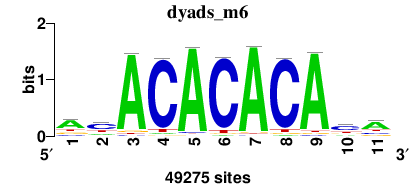 | no match | 0 |
| dyads_m7 | 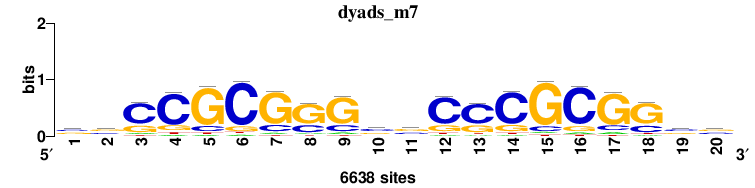 | no match | 0 |
| dyads_m8 | 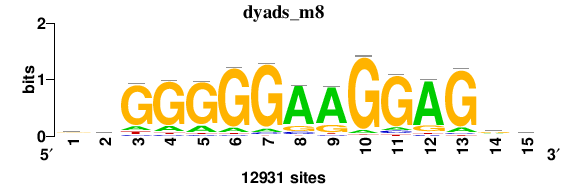 | MZF1(var.2) | 0 |
| dyads_m9 | 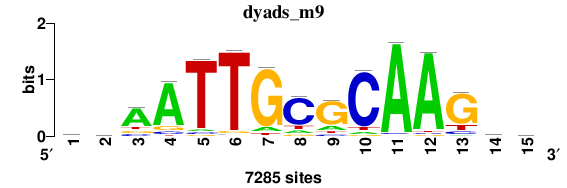 | CEBPE, CEBPB, CEBPD | 0 |
| dyads_m10 | 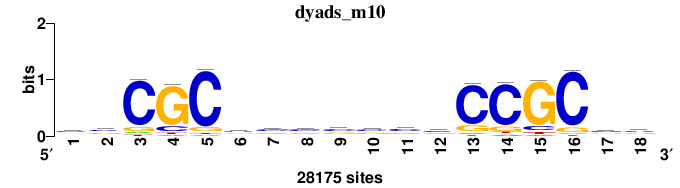 | no match | 0 |

**Supplementary Table 2: List of enriched *de novo* motifs within C/EBPε peaks.** Analysis of *de novo* motif enrichment performed by RSAT using oligo-analysis, position-analysis, local-word-analysis and dyad-analysis algorithms with oligomer lengths ranging from 6 to 8 across C/EBPε binding sites shows a significant enrichment for C/EBP motif (marked by a red asterisk) as well an enrichment for a potential C/EBP-AP1 heterodimer motif (marked by two red asterisks).
